# Patients' perspectives on high-tech home care: a qualitative inquiry into the user-friendliness of four technologies

**DOI:** 10.1186/1472-6963-4-28

**Published:** 2004-10-05

**Authors:** Pascale Lehoux

**Affiliations:** 1 Department of Health Administration, Interdisciplinary Health Research Group (GRIS), University of Montreal, P.O. Box 6128, Branch "Centre-ville", Montreal, Quebec H3C 3J7 Canada

## Abstract

**Background:**

The delivery of technology-enhanced home care is growing in most industrialized countries. The objective of our study was to document, from the patient's perspective, how the level of user-friendliness of medical technology influences its integration into the private and social lives of patients. Understanding what makes a technology user-friendly should help improve the design of home care services.

**Methods:**

Four home care interventions that are frequently used and vary in their technical and clinical features were selected: Antibiotic intravenous therapy, parenteral nutrition, peritoneal dialysis and oxygen therapy. Our qualitative study relied on the triangulation of three sources of data: 1) interviews with patients (n = 16); 2) interviews with carers (n = 6); and 3) direct observation of nursing visits of a different set of patients (n = 16). Participants of varying socioeconomic status were recruited through primary care organizations and hospitals that deliver home care within 100 km of Montreal, the largest urban area in the province of Quebec, Canada.

**Results:**

The four interventions have *both *a negative and positive effect on patients' lives. These technologies were rarely perceived as user-friendly, and user-acceptance was closely linked to user-competence. Compared with acute I.V. patients, who tended to be passive, chronic patients seemed keener to master technical aspects. While some of the technical and human barriers were managed well in the home setting, engaging in the social world was more problematic. Most patients found it difficult to maintain a regular job because of the high frequency of treatment, while some carers found their autonomy and social lives restricted. Patients also tended to withdraw from social activities because of social stigmatization and technical barriers.

**Conclusions:**

While technology contributes to improving the patients' health, it also imposes significant constraints on their lives. Policies aimed at developing home care must clearly integrate principles and resources supporting the appropriate use of technology. Close monitoring of patients should be part of all technology-enhanced home care programs.

## Background

The possibility of managing patients outside of the hospital has rarely been so widely considered. Indeed, home care is often seen as less costly and more patient-friendly [1]. However, the effectiveness and safety of home care have yet to be subjected to rigorous study [2,3]. The transfer of care from the hospital to the home raises economic and organizational issues [2]. For example, low levels of physician involvement [4] and significant increases in private spending [5] have been observed. Furthermore, the impact of the increasing use of technology in home care has not been examined [6]. A recent survey showed that 98% of Quebec (Canada) primary care organizations are using programmable pumps to deliver intravenous antibiotics therapy at home, and 84% are providing home oxygen therapy [7]. The growth of technology in home care means that lay people with varying levels of technical skills and education become direct users of health technology [6]. Therefore, the objective of our study was to document, from the patient's perspective, how the level of user-friendliness of medical technology influences its integration into the private and social lives of patients. More specifically, we adopted a "technology-in-practice perspective", which relies on qualitative in-depth investigation, by looking at what technologies do and help accomplish in the daily practices of technology users and in the organization of health care [8].

Sullivan [9] showed how growing interest in the patient's perspective is the result of convergent trends in health and social scientific research. There is a growing appreciation of how the patients' values affect their experience of a chronic health state. Medical sociologists have shown that shifting family and social relationships shape patients' perceptions and coping strategies [10]. For instance, Lowton and Gabe [11] observed that adults with cystic fibrosis, who were not expected to live for long, deployed diverse strategies to downplay the importance of their illness and compare themselves favourably to "normal, healthy" people. Although clinicians are often concerned about patient compliance, the technical and human dimensions at the root of this problem remain understudied [10,12]. Observers of high-tech home care have stressed that certain health technologies are, by their very design, unfriendly [6]. Nevertheless, very little is known about the characteristics that facilitate or impede the use of medical devices and whether patients perceive them as user-friendly or not.

The term user-friendly is used to characterize an object – often a computer system – as "easy to operate or understand; not needing special training" [13]. This notion has gained impetus over the last 20 years with the growth of information technology and research into Human-Computer Interface (HCI). In general, the human-machine interface is seen as key in enabling a smooth fit between the user, the task and the technology [14,15]. The need to design interfaces that users can rapidly understand and interact with was recognized several decades ago as it impacted workers' efficiency. Although the "overriding ethos within the community of system designers has been to try to ensure that the system is user-friendly," it remains difficult for designers to grasp all of the subtleties that shape users' needs and practices [[15]: p.126]. According to Norman, "There is a big difference between the expertise required to be a designer and that required to be a user. In their work, designers often become expert with the *device *they are designing. Users are often expert at the *task *they are trying to perform with the device" [[16]: p.156].

In the field of health care, this specialization often adds to the complexity of the work of designers, who, in addition to not being end users of the device, are simply unfamiliar with the complicated tasks health care providers are achieving through the use of technology. When moving health technologies away from the hospital and into the patient's home, the design characteristics of medical devices become even more salient, since patients have to learn how to operate them safely and with confidence. According to Norman [16], the use of any device is learned more readily if the user has a good conceptual model. "This requires that the principles of operation be observable, that all actions be consistent with the conceptual model, and that the visible parts of the device reflect the current state of the device in a way consistent with that model" [[16]: p.189]. Hence, the designer must create a conceptual model that is understandable for the user and that captures the important steps of the operation of the device. In a similar perspective, when assessing the level of user-friendliness, Lun [14] has suggested paying attention to two components: 1) user-acceptance – the extent to which the user is favourable to using the technology; and 2) user-competence – the abilities required to use the technology effectively. These two components interact with each other – the more technically complex a technology, the more elaborate the user training required. This author also underlined three principles for designing user-friendly interfaces: 1) human-machine interaction is pivotal; 2) this interaction evolves through use; and 3) user should be the key informant. Methodologically speaking, these principles imply that designers should compile users' perspectives, directly observe how technology is being used, and identify the learning curve by which technology is appropriated by users.

Because most computer interfaces are used in a somewhat confined environment, the work of scholars who have studied technical aids for the disabled and the elderly brings another dimension to the definition of user-friendliness. Conceptualizing disability as a social phenomenon, the user-friendliness of technical aids has to be gauged with respect to their ability to assist users' move freely in their social environment [17]. From this perspective, autonomy and mobility become prominent, as well as the impact of the technology on users' social identity. For instance, Pippin and Fernie [18] conducted focus groups and interviews with elderly patients in order to explore the following issues: Acceptance of dependence, experience of social stigma, recognition of one's own physical loss, appearance of technical aids, autonomy and perceptions of alternatives. This work shows that users have to cope not only with the technology but, more importantly, with the limitations they themselves experience (i.e., architectural barriers, growing old) and that may become the object of others' gazes as soon as they engage in environments outside of their private sphere. Thus, the importance of user-acceptance and user-competence will vary depending on where the technology is used. The level of user-friendliness would then be a function the type of settings in which users circulate, which affects how they succeed in (re)constructing their identity as a medical technology-user.

Social scientific work on patients learning how to cope with chronic illnesses and life-sustaining technology has offered similar observations, highlighting that each patient tends to go through a personal trajectory, or engage in "biographical work" whose aim is to give meaning to a constellation of unfolding events [10]. Here also, the setting where services are provided is likely to influence patients' perceptions and coping strategies. Although the hospital enables the patient to adopt the sick role rather straightforwardly, the home setting may force him/her to be more active and show optimism [5,6]. Family members and caregivers are also affected by the use of high-tech home care. They might be asked to provide technical and moral assistance, while coping with a profoundly modified family dynamic. In certain cases, providing assistance implies inflicting pain and discomfort [6].

**Figure 1:**
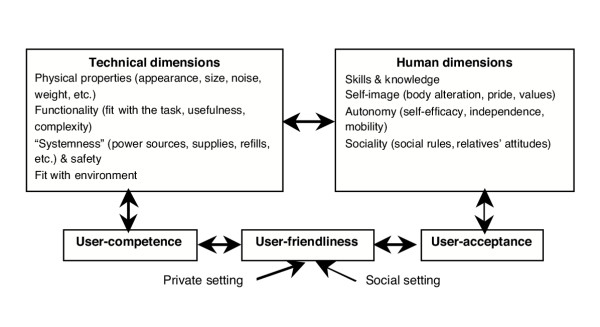
Technical and human dimensions that shape the user-friendliness of a technology (drawing on 12 and 13)

To summarize, the literature underscores the many technical (e.g. weight, functionality, complexity) and human (e.g. self-image, cognitive resources, social stigma, pain, etc.) variables that influence the use of technology and which are affected by the setting (institutional, private or public) where technology use takes place. The user-friendliness of a technology therefore results from a smooth fit between human and technical features, with the fit varying between and within settings and individuals. Figure 1 illustrates the relationships between these variables. This framework posits that technical dimensions largely influence user-acceptance, and human dimensions will affect user-competence. In addition, the level of autonomy that a technology can provide in private and social settings is both shaped by its technical features and the human factors associated with its use. Accordingly, this study sought to define, from the patient's perspective, the extent to which different home care interventions could be considered user-friendly and how they were integrated into patients' private and social lives.

## Methods

We selected four interventions: Antibiotic intravenous therapy (IV), parenteral nutrition (PN), oxygen therapy (O2) and peritoneal dialysis (PD). These were chosen because they are frequently used [7] and vary in their technical and clinical features, and so are likely to differentially influence how users interact on a daily basis with them (see Table 1).

Our study relied on the triangulation of three sources of data. We conducted semi-directed, individual interviews with patients (n = 16) and carers (n = 6), and directly observed nursing visits, involving a different set of patients (n = 16). This strategy enabled us to gather data on a broader set of patients. The carers we interviewed were not necessarily related to a patient participating to our study (to reduce pressures on interviewees) and were spouses or family members (often mother or daughter) of a person receiving high-tech home care. Our purposeful sampling strategy was to diversify viewpoints, by including participants of varying socioeconomic status, gender and age (Table 2). These variables are all likely to affect how patients and their carers adapt to the use of technology (e.g. contracting out private home care services, adapting the home, understanding written instructions, etc.). All participants were recruited through primary care organizations (for IV and O2) or hospital-based home care programs (for PN and PD) located within 100 km of Montreal, the largest urban centre in the province of Quebec (Canada). A member of the nursing staff in these organizations was asked to give a brochure to all eligible patients explaining the objectives and procedures of the study. After contact had been established between patients and our research team, we constructed the sample according to our diversification variables. We obtained approval from the organizations' ethics committees.

Our approach was structured according to symbolic interactionism, which focuses on how individuals, through regular interactions, develop shared meanings and conceptualize, perceive and understand the role of technology [[19]: p.201]. This approach was particularly helpful in identifying how patients, formal caregivers and informal carers, through their experience in interacting together, anticipated and defined the contributions and responsibilities of each other. Interviews were biographical, relying on Lafaille and Lebeer's technique for examining coping strategies [20]. Interview questionnaires were structured to systematically explore the themes highlighted by our framework while allowing the interviewee to develop or introduce issues he/she felt were important (questionnaire available upon request). Carers' interviews were key to eliciting how they, themselves, perceive the technology – they often have to intervene when the patient is tired or not feeling well – and how the patients' social lives were transformed because of the use of technology. Interviews lasted 60–120 minutes, and were audio-taped with the written consent of the interviewee, then transcribed into electronic format. Direct observations enabled a better understanding of how patients were educated about, and supported in the use of technology [21]. An observation guide was used to rapidly record descriptive notes during the visit, while a structured summary of key events was written up subsequent to the visit [22]. The NUD*IST software was used to code and selectively retrieve verbatim extracts [23]. A mixed strategy was applied: Codes were either derived from our framework or created when their recurrence across interviews became significant. Our analyses were designed to compare and contrast the participants' experiences with using technology, both inside and outside the patient's home. We drew up tables [24], summarizing the observations stemming from the three sources of data, to identify the main technical and human factors at play in private and social settings for each of the four technologies. Most verbatim extracts were translated from French to English, then slightly edited for the purposes of this paper.

## Results

Home care technology transforms the patient's life both inside and outside the home

### In the patient's home

User-acceptance was shaped by different types of anxiety (see Table 3). In the case of IV and PN, the catheter access site must be protected to avoid potential infections and dislodging of the catheter. The alarm system of the programmable pump, used for both interventions, tends to go off too easily (e.g. occlusions when the tube gets twisted). These false alarms were initially perceived as very stressful but, over time, they became a "normal" disturbance: "I don't really sleep at night. I'm afraid the catheter will get dislodged and the alarm will go off" (Interview, PN, w3). O2 patients were concerned about the risk of fire when cooking over a gas stove or being in a room with smokers. PD patients were preoccupied by a demanding regimen that required them to balance treatment with meals and other daily activities. Nevertheless, some enjoyed being empowered through greater involvement: "You do all the follow-up yourself: Why is my blood pressure high today? Do I have edema?" (Interview, PD, m2). User-competence was affected by the relationship between patient and carer. One PD patient felt annoyed by his wife who, at the beginning, was checking whether he was applying the aseptic procedures rigorously (Obs., PD, m1). The opposite was observed for a PN patient, whose partner felt useless and avoided her during the treatments (Obs., PN, w3).

The IV patients' perceptions of the technical aspects of their technology were striking. With use of this technology being, in most cases, temporary, patients were generally passive or even submissive: "You're always a slave to it, having to carry it everywhere" (Interview, IV therapy, w2). User-acceptance is in fact closely linked to competence. Older patients on IV did not feel comfortable with the electronic components of the programmable pump, which they associated with the "computer age" and about which they felt ignorant. Chronic patients seemed, in general, keener to master the technical aspects. "You can't live without air! So you have to be careful and do it right" (Interview, O2, carer, m1). One PN patient was technically confident and had developed her own technique for preventing air bubbles from forming in the tube (Obs., PN, w1). A similar confidence was shown by the carer of a PD patient: "When you see all that stuff – the reservoir, the wires – you wonder if you'll be able to do it! But, once you know how, it's easy" (Interview, PD, carer, w1). For all four interventions, manual dexterity was required to properly manipulate the different components. "If my eyes were okay, I'd have been able to do it. But I was frightened of not doing it properly, of not seeing the needle, which is so tiny" (Interview, IV, carer, w1). We also observed patients who were not able to read messages on the digital screen due to poor eyesight (due to old age or co-morbidity), limited English linguistic skills, or illiteracy. They relied on their memory or made informed guesses when operating the device.

Finally, the technology did not always fit neatly in the home setting: "Well, you wouldn't believe how hot [the room gets] when the door is closed!" (Interview, O2, caregiver, m3). Some O2 patients liked to have an extra set of tubing so they could use a second floor or sit outside on a patio (Obs., O2, w1). One PD patient planned to have an evacuation system installed so he would not have to dispose of the solution exiting the tubing from his peritoneal cavity through the toilet anymore (Obs., DP, m1).

### In the patient's social life

While some of the barriers described above can be managed fairly well in the home setting, problems arise in the unpredictable "outside world": "It's great when you're at home where you're all set up. But when you're out, you're always worried about people lighting up a cigarette" (Interview, O2, carer, m3) (see Table 4). In addition, O2 patients did not like to be seen with nasal tubes, and less often invited friends over or ate in restaurants (Obs., O2, w1). In the case of PN patients, who rarely or never eat food, it was relatives who felt uncomfortable and tended to invite them over less often. Carers sometimes curtailed their social activities because they felt needed by the patient: "I didn't dare go out, absolutely not" (Interview, IV, carer, w1). The mother of a woman with PN was "always worried" and "always available" (Interview, PN, carer, w1). A wife "found the manual PD a burden – four times a day... It's like being in jail, you can't go anywhere" (Interview, PD, carer, w1).

The non-retired patients experienced major obstacles in continuing with employment because of the frequency and/or duration of treatments. Few of these patients had a full-time job. Being "hooked up" to a fixed O2 concentrator for up to 18 hours/day is not compatible with many types of work, and portable cylinders also restrict autonomy (2–4 hours). One PN patient said it was "impossible to work because of being connected for 12 hours", and receiving lots of fluid at night compromised her sleep (Interview, PN, w2). On the other hand, one PD patient chose a nocturnal exchange regulator, refusing hospital-based hemodialysis, in order to work: "You can't work if you go to the hospital three times a week, and work is very important to me" (Interview, PD, m2). In the latter case, the comparison with an alternative makes PD more acceptable.

More crucial was the "black-and-white" definition of ability to work that governs disability pension plans, which is incompatible with what patients experience: "You know, sometimes you feel okay, and sometimes you don't; sometimes you're disabled and sometimes you're not..." (Interview, PN, w3). Overall, due to compromised health and technical barriers, most patients agreed to apply for a disability plan (according to which they cannot accept paid work). A number of patients did volunteer work, such as helping neighbourhood kids with their homework (Obs., PN, w1), doing clerical work for the family business (Obs., O2, w1), or volunteer work for a patient association (Obs., O2, w3).

## Discussion

This study, adopting a "technology-in-practice" perspective [8], shows that the four interventions have *both *a negative and a positive effect on the lives of patients and carers. Indeed, our analyses sought to provide more detail on how technology simultaneously improves and constrains patients' lives. This is compatible with Pierret's observation that "Medicine gives the chronically ill reason to hope, even as it produces limitations with which these persons have to live by making adjustments to meet everyday requirements" [[12]: p.14]. Although each technology provided patients with relative autonomy from the hospital, none of them were seen by patients as truly user-friendly. IV patients remained passive and accepting, knowing that the constraints were temporary, and although O2 does not require a high level of competence, user-acceptance remained very low, especially in public places. Patients seemed more likely to develop competence in using both PN and PD because alternatives in these cases are limited (e.g. hospital-based services or death) and acceptance becomes the only way to make sense of this whole (life-long) experience. Such findings highlight the need to increase the fit between users and technology through a better design of high-tech home care devices *and *through effective patient education strategies. Indeed, competence and acceptance are likely to be mutually reinforced, especially if patients are supported and their know-how re-assessed over time. Although this is already part of the nursing staff duties, the experiences shared by our interviewees and the literature [1,2] indicate that the level of support they receive may be insufficient.

As observed in a recent U.K. survey on the quality of primary health care provision, crucial factors include educational training, patient education programs and improved communication and teamwork [25]. In the case of home care, manifesting a greater concern with supporting patient education is particularly relevant in the current health policy context [1,5], where high-tech home care is increasingly seen as an "easy solution" to budgetary constraints and a growing elderly population. However, this study pinpoints the possibility that using high-tech home care without a proper patient support system might create more problems than it would solve. As stressed by Sinding, "It is only in more collectively oriented social action that higher standards of care can be established (or re-established) as within the purview of health professionals' duties, and thus confirmed as patients', and carers', entitlements" [[26]: p.1384]. It is in this perspective that this study's results take on significance.

In particular, two issues related to policy-making and clinical practice require prompt consideration. First, technology is often designed as though patients all possess similar abilities, and are neither ageing or incapacitated by other illnesses or physical disabilities [18]. Since the development of home care is largely industry-driven [6], technology designers should be asked to gather and more explicitly integrate feedback from different groups of users with varying cognitive and physical capacities [27,28]. Norman [16] suggests paying attention to two components that affect the use of technology – the design model and the user's model. Both are more or less implicit explanations (or "road maps") about how to operate the device. When the gap between these two models becomes too significant, misuse – which can lead to ineffective treatments or harmful consequences – is likely to happen. Since the biomedical equipment designer and the patient rarely, if ever, interact, it is critical that the proper use of the technology be "communicated" through its physical appearance, by the way it responds (visual and/or audio feedback), and by its fit with the private and social settings where patients are evolving.

Second, competence of patients and their carers should be reinforced through adequate education and support from physicians and nurses. Medical specialists who manage hospital-based home care programs for O2, PN, PD patients could emphasize, when enrolling patients, the need for a smooth fit between the technical and human barriers that affect patient compliance. Training programs for nurses could also focus on skills and routines that help increase the user-friendliness of technology [29,30]. Finally, both patients and clinicians need to be involved in redesigning home care services so they meet the diverse and changing needs of chronic patients [[28]: p.877].

This study has sought to better understand how the level of user-friendliness of medical technology influences its integration into the private and social lives of patients. In this regard, qualitative methods are particularly well suited for uncovering patients' views. Nevertheless, the limitations of our study should be acknowledged [21,31]. The reason for including four different interventions was to define which technical and human dimensions make health technology user-friendly (or not). This study design characteristic increased the complexity of the sampling strategy. For instance, we could not explore the specific role of variables such as gender, age and ethnicity [28]. The study design, however, put a broader perspective on the research problem. Indeed, redundancy from one interview to another and the growing saturation of our analytical categories suggest that we have captured the key elements associated with the introduction of high-tech home care into patients' lives. Overall, the triangulation of three data sources increased the credibility (or internal validity) of our findings by sharpening our understanding of how technologies are integrated into patients' lives. Our findings should, therefore, be applicable in countries and regions where similar devices are used and in similar home settings [21,31]. Finally, although lower levels of criticism were to be expected, thereby reflecting positive functional avoidance [26], overall, the participants we interviewed expressed several grievances. This is similar to the findings of a recent qualitative study where patients, in retrospect, "regretted accepting, in hope, the offer of 'active treatment' because of reduced quality of life" [[28]: p.4]. Because home care patients may, over time, adopt less than optimal routines, the concept of "acceptance" thus requires careful analysis and further investigation. There is a danger of forgetting about the "social and political factors that sustain perceptions of health system constraints" as being unavoidable, and therefore acceptable [[26]: p.1384].

## Conclusion

This paper shows that the barriers facing home care patients can easily be "taken for granted," as though nothing can be done to improve the situation. With a growing elderly population and limited health care resources, this will become a major issue in most industrialized countries. According to McGarry, "The home environment as a location of care provision is largely beyond the public-professional gaze, and therefore, remains potentially hidden from scrutiny" [[32]: p.429]. In the future, the delivery of high-tech home care is likely to grow [1]. Nonetheless, this study indicates that patients who are asked to become users of medical technology face major challenges. As stressed by Sullivan, moving beyond a discussion of the benefits of technology to patients' health, to a consideration of *both *the positive and negative impact of technology on patients' lives, not only brings medicine closer to issues that really matter to patients, but also generates "greater scientific, ethical, and social complexity" [[9]: p.1602]. Home care involves more than simply transferring a particular technology from the hospital to the home – it requires transferring knowledge and skills to lay people, and making sure that the home and social environments enable a safe, effective, appropriate and personally satisfying use of technology. Otherwise, ineffective, potentially hazardous and socially compromising treatments may be disseminated. Policies aimed at increasing the provision of home care must carefully integrate principles and resources that support the appropriate use of technology, and close monitoring of patients must be part of all technology-enhanced home care programs [3].

## Competing interests

The author declares that she has no competing interests.

## Pre-publication history

The pre-publication history for this paper can be accessed here:



## Figures and Tables

**Table 1:** Description of the four interventions

**IV therapy**:	Used on a short-term basis to treat severe infections. The antibiotic drug can be delivered via a catheter in the patient's vein using different mechanical or electronic devices. Programmable pumps, which are battery-powered and can be carried in a shoulder bag, are seen as more reliable. Pumps are equipped with an alarm that warns of an occlusion, inadequate connection of tubes, or low battery.
**Parenteral nutrition (PN):**	Used when oral nutrition is no longer feasible due to a disease (e.g. Crohn's disease, cancer of the digestive tract). Fluids and nutritive solutions are delivered via the patient's vein with the help of a programmable pump. A catheter is surgically inserted on a long-term basis. Patients are required to comply with very strict aseptic procedures. Patients generally use the device every day, and it may take up to 8 hours (overnight) to deliver the required amount of solution.

**Peritoneal dialysis (PD):**	An alternative to hospital-based hemodialysis that is designed to remove urea from the blood. It also involves the insertion of a permanent catheter, in this case through the peritoneal cavity, which requires compliance with aseptic procedures. A liquid is inserted in the peritoneal cavity and flushed out. Using an electronic device, patients can set automated exchanges to occur overnight, but when using a gravity system, patients have to manually perform 3 to 5 exchanges per day.

**Oxygen therapy (O2):**	Prescribed to patients with severe hypoxemia (due to pulmonary dysfunction). Oxygen is delivered by a fixed concentrator, via a 15-meter tube and nasal device. Patients normally use it up to 18 hours a day. Portable cylinders may be used for short periods (2–4 hours). A small oxygen-saving device allows oxygen to be delivered only when the patient is inspiring.

**Table 2:** Details about interviewees

	**Patients**			**Carers**	
**Intervention**	**Gender**	**Age**	**Length of use **(months)	**Gender**	**Age**

**IV therapy**	2 women	38/80	24/1	1 woman	67
	3 men	65/65/68	2/1.5/1.5		

**Parenteral nutrition**	5 women	29/45/45/ 50/62	120/24/36 84/18	1 woman	72

**Oxygen-therapy**	3 women	48/62/82	96/24/36	1 man	83

**Peritoneal dialysis**	3 men	25/48/51	24/12/12	2 women 1 man	35/45 70

Summary	10 women + 6 men = 16	53.9 (25–81)	IV: 6 Others: 44.2	4 women + 2 men = 6	62

**Table 3:** Technical and human factors that affect how patients use health technology at home

	**IV therapy (IV)**	**Parenteral nutrition (PN)**	**Oxygen therapy (O2)**	**Peritoneal dialysis (PD)**
**Technical factors**	Relatively few manipulations are required, but these may become problematic for older people lacking fine manual dexterity The alarm system of the programmable pump frequently disturbs sleep	Technical tasks are numerous and require dexterity The alarm system of the programmable pump frequently disturbs sleep Aseptic procedures are crucial Storage space is required for feeding solutions (extra fridge) and durables The gravity pole bumps against carpet edges and door ledges, creating bubbles in the bag	Technical tasks are simple (changing filters, connecting tubes to cylinders) Concentrator generates noise and heat, while 15-metre long tubes run along the floor	Technical tasks are numerous and require dexterity The bedroom resembles an hospital room, and waste solutions have to be emptied into the toilet

**Human factors**	Anxiety is triggered by the possibility of the catheter becoming dislodged Daily activities such as cooking or taking care of a child are compromised	Anxiety is triggered by the possibility of the catheter becoming dislodged Women feel their body is not attractive anymore because of the catheter Patients attempt to hide medical equipment from the eyes of visitors	Anxiety is triggered by the dangers associated with getting too close to smokers or flames Daily activities such as cooking or taking care of a child are compromised	Patients are struggling to control their health by balancing their dietary regimen and treatments The permanent catheter alters the patient's body image

**Table 4:** Technical and human factors that affect how patients use health technology in broader social life

	**IV therapy (IV)**	**Parenteral nutrition (PN)**	**Oxygen therapy (O2)**	**Peritoneal dialysis (PD)**
**Technical factors**	Portable systems may be heavy and limit mobility, while the gravity pole confines the patient to the home	Enables patients to be independent of the hospital and clinical staff Treatment frequency is a major constraint	Patients are confined to a restricted space ("hooked up" to tubes) The portable cylinders provide short periods of autonomy (2–4 hours)	The nocturnal automated exchange regulator enables a certain level of autonomy Treatment frequency is a major constraint

**Human factors**	Professional and social life is slightly limited, albeit for a short period	Professional life is limited because of treatment frequency, the disease itself and the occasional-to-frequent re-hospitalisations Social life is limited because so much of social life revolves around the sharing of meals	Professional life is limited because of the disease and being "hooked up" to the concentrator for up to 18 hours/day Social life is limited because of the compromised self-image associated with wearing nasal tubes and portable cylinders The oxygen-saving device generates noise similar to that of a ventilator	Professional life is still possible (and is less restricted than for patients on hospital-based hemodialysis) Social life is still possible when using the nocturnal exchange regulator, but complicated when one wants to travel abroad or make short trips
